# Effect Of Cariprazine On Metabolic Parameters In Patients Living With Bipolar Disorder: A Systematic Review And Meta-Analysis

**DOI:** 10.1192/j.eurpsy.2026.10427

**Published:** 2026-07-31

**Authors:** M. Pompili, M. Cifrodelli, A. V. Mammoliti, F. Formica, R. Iannazzo, R. McIntyre, I. Berardelli

**Affiliations:** 1 Department of Neurosciences, Mental Health and Sensory Organs, Faculty of Medicine and Psychology, Suicide Prevention Centre, Sant’Andrea Hospital, Sapienza University of Rome, Via di Grottarossa, 1035, 00189; 2 Department of Human Neurosciences, Sapienza University of Rome; 3 Psychiatry Residency Training Program, Faculty of Medicine and Psychology, Sapienza University of Rome, Sant’Andrea Hospital, 00185, Rome, Italy; 4 Department of Psychiatry, University of Toronto, Department of Pharmacology and Toxicology, University of Toronto, Toronto, Ontario, Canada

## Abstract

**Introduction:**

Cariprazine is a second-generation antipsychotic approved for treating schizophrenia, acute manic or mixed episodes associated with bipolar I disorder and as adjunctive treatment in major depressive disorder. Antipsychotic treatment is often associated with metabolic alterations including weight gain, dyslipidemia, and increased risk of type 2 diabetes and cardiovascular disease.

**Objectives:**

We performed a systematic review and meta-analysis with the overarching aim of synthesizing study results that describe the effect of cariprazine on glucose and lipid homeostasis as well as weight in persons living with bipolar disorder.

**Methods:**

Six meta-analyses focused on patients with bipolar disorder (BD), evaluating changes in weight, cholesterol, and glucose for both Cariprazine 1.5 mg and 3 mg.

**Results:**

Cariprazine showed a limited impact on the metabolic profile in BD. Particularly, weight gain was statistically significant in both doses, but clinically limited (<1 kg), with a percentage of subjects reporting an increase ≥7% of body weight less than 5%, confirming the relative metabolic safety of the drug also in this population.

**Conclusions:**

This meta-analysis suggests that cariprazine, although associated with modest weight gain, without significant alterations in lipid and glycemic profiles, shows a favorable metabolic profile compared to other atypical antipsychotics.

**Disclosure of Interest:**

None Declared

